# Inhibiting histone deacetylases suppresses glucose metabolism and hepatocellular carcinoma growth by restoring FBP1 expression

**DOI:** 10.1038/srep43864

**Published:** 2017-03-06

**Authors:** Jing Yang, Xin Jin, Yuqian Yan, Yingjie Shao, Yunqian Pan, Lewis R. Roberts, Jun Zhang, Haojie Huang, Jingting Jiang

**Affiliations:** 1 Department of Tumor Biological Treatment, The Third Affiliated Hospital of Soochow University, 185 Juqian Street, Changzhou, 213003, China; 2 Department of Biochemistry and Molecular Biology, Mayo Clinic College of Medicine, Rochester, MN, 55905, USA; 3 Jiangsu Engineering Research Center for Tumor Immunotherapy, Changzhou, Jiangsu, 213003, China; 4 Division of Gastroenterology and Hepatology, Mayo Clinic College of Medicine, Rochester, MN, USA; 5 Department of Laboratory Medicine and Pathology, Mayo Clinic College of Medicine, Rochester, MN, 55905, USA; 6 Mayo Clinic Cancer Center, Mayo Clinic College of Medicine, Rochester, MN, 55905, USA

## Abstract

Hepatocellular carcinoma (HCC) is one of the most commonly diagnosed cancers in the world. Elevated glucose metabolism in the availability of oxygen, a phenomenon called the Warburg effect, is important for cancer cell growth. Fructose-1,6-bisphosphatase (FBP1) is a rate-limiting enzyme in gluconeogenesis and is frequently lost in various types of cancer. Here, we demonstrated that expression of FBP1 was downregulated in HCC patient specimens and decreased expression of FBP1 associated with poor prognosis. Low expression of FBP1 correlated with high levels of histone deacetylase 1 (HDAC1) and HDAC2 proteins in HCC patient tissues. Treatment of HCC cells with HDAC inhibitors or knockdown of HDAC1 and/or HDAC2 restored FBP1 expression and inhibited HCC cell growth. HDAC-mediated suppression of FBP1 expression correlated with decreased histone H3 lysine 27 acetylation (H3K27Ac) in the *FBP1* enhancer. Restored expression of FBP1 decreased glucose reduction and lactate secretion and inhibited HCC cell growth *in vitro* and tumor growth in mice. Our data reveal that loss of FBP1 due to histone deacetylation associates with poor prognosis of HCC and restored FBP1 expression by HDAC inhibitors suppresses HCC growth. Our findings suggest that repression of FBP1 by HDACs has important implications for HCC prognosis and treatment.

The Warburg hypothesis highlights that tumor cells metabolize glucose into lactate even in the presence of high oxygen. This metabolic alteration is proposed to be one of the fundamental causes of cancer^1^. Activation of oncogenes and mutations in tumor suppressor genes are known to be responsible for the Warburg effect in tumors. For example, AKT1 stimulates glycolysis by increasing the expression and membrane translocation of glucose transporters, and by phosphorylating key glycolytic enzymes such as hexokinase and phosphofructokinase-2^2^,^3^. The p53 protein inhibits the glycolytic pathway by up-regulating the expression of TP53-induced glycolysis and apoptosis regulator (TIGAR)^4^. In recent years, scientific community has paid more attention to the aerobic glycolytic pathway. Gluconeogenesis is the major component regulating glucose homeostasis and has also been suggested to play an equally important role in switching of tumor cells towards aerobic glycolysis^5^. FBP1 is a rate-limiting enzyme in gluconeogenesis, and its loss seems to be a critical oncogenic event in epithelial-mesenchymal transition-promoted basal-like breast cancer cell progression^5^. Expression of FBP1 is downregulated in gastric and colon cancer cells^6^,^7^, and its loss associates with poor prognosis of clear cell renal cell carcinoma^8^. This suggests that FBP1 plays an important role in modulating glucose metabolism in cancer and is associated with cancer development and progression.

Epigenetic changes including histone modifications and DNA methylation have been shown to alter the pattern of gene expression, resulting in various pathological conditions including cancer^9^. Histone modifications such as acetylation and methylation play an important role in regulation of gene transcription. The overall level of histone acetylation is regulated by the activity of various enzymes including HDACs, which are involved in histone deacetylation and influence the affinity binding of histone proteins with the DNA backbone. HDACs have been observed to be overexpressed in several cancer types^10^,^11^,^12^.

HCC is one of the most common malignant diseases in the world and often associated with poor prognosis. Its incidence rate is increasing, especially in Asian countries due to higher rate of HBV and HCV infections^13^. Since deregulated glucose homeostasis plays an important role in the occurrence and development of tumors, in the present study we attempted to analyze the expression of FBP1 in HCC cells in culture and in patient specimens and determine the molecular mechanisms underlying its deregulation and its role in HCC cell growth.

## Results

### Decreased FBP1 expression in HCC tissues correlates with poor prognosis

**Figure 1:**
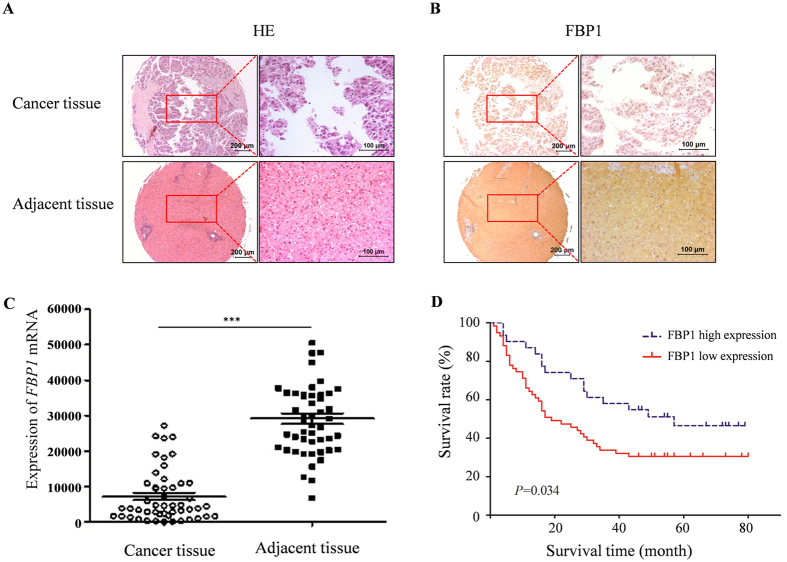
Analysis of FBP1 expression in HCC and its correlation with patients’ survival. (**A**,**B**), Haematoxylin and eosin (H&E) staining (**A**) and FBP1 immunohistochemistry (**B**) of HCC tissues and adjacent benign tissue samples. (**C**) Expression of *FBP1* mRNA in HCC and adjacent benign tissues determined by meta-analysis of the TCGA dataset. (**D**) Kaplan-Meier plots showing the association of low expression of FBP1 with overall survival of HCC patients (ninety cases in total, 33 cases with high expression of FBP1, and 57 cases with low expression of FBP1).

We first analyzed FBP1 expression in tissue microarray (TMA) consisting of HCC specimens and adjacent benign tissues using immunohistochemistry (IHC). We found that FBP1 expression was detected in the cytoplasm and nucleus of cells in benign tissues, but the level of FBP1 protein was much lower in approximately 66% of cancerous tissues examined (Z = 7.952, *P* < 0.001). Representative images of haematoxylin and eosin (H&E) staining and FBP1 IHC in HCC and adjacent benign tissues are shown in Fig. 1A and B, respectively. Meta-analysis of The Cancer Genome Atlas (TCGA) dataset showed that FBP1 mRNA levels were significantly lower in HCC tissues compared to adjacent benign tissues (Fig. 1C). To further interrogate the relationship between FBP1 expression and clinicopathological parameters, patient samples were categorized into different groups based on sex, age, tumor size, pathological grade, AJCC stage and cirrhosis. As shown in Table 1, groups with low and high FBP1 expression differed significantly with respect to the tumor size (*P* = 0.005) and AJCC stage (*P* = 0.001). In addition, we also analyzed the association of FBP1 with overall survival using Kaplan-Meier and Log-rank test. We demonstrated that low expression of FBP1 associated with poor survival of patients (Fig. 1D). Moreover, we found that the mean survival time of the group with high FBP1 expression was 50.17 months, but was 34.88 months in the low expression group (*P* = 0.034). As illustrated in Table 2, the cumulative survival rate of the FBP1 high expression group was much longer than that of the FBP1 low expression group. Thus, these data indicate that low expression of FBP1 in HCC tissues correlates with poor prognosis.

### HDAC inhibitors induce derepression of FBP1 mRNA and protein levels in HCC cells

**Figure 2:**
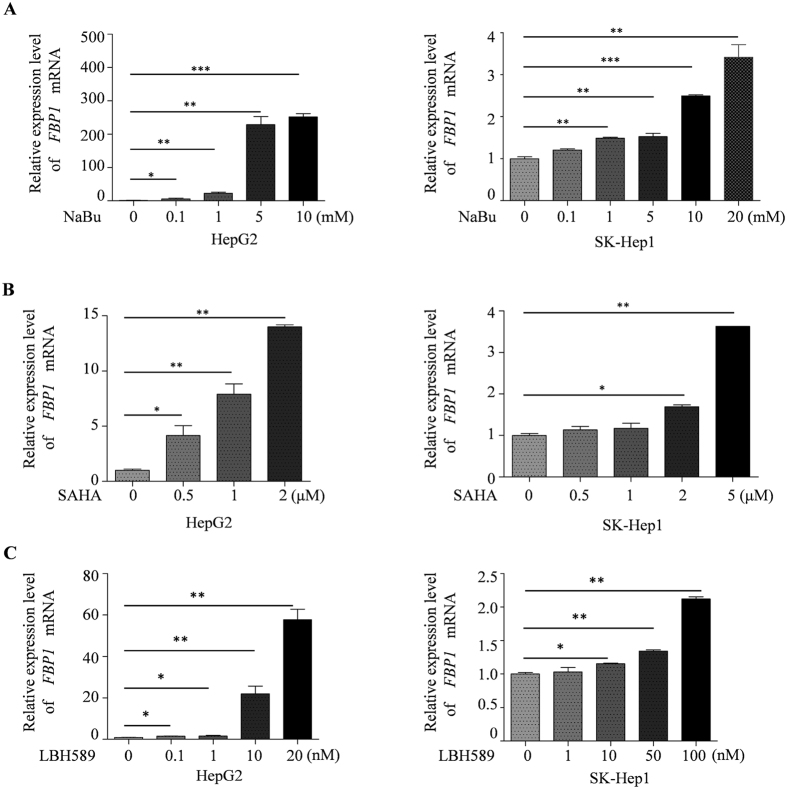
Effects of HDAC inhibitors on *FBP1* mRNA expression in HCC cells. HepG2 and SK-Hep1 cells were treated with different dosages of HDAC inhibitors including NaBu (**A**), SAHA (**B**) and LBH589 (**C**) for 24 hours. Cells were harvested and *FBP1* mRNA expression was analyzed by real-time RT-PCR and *GAPDH* was used as an internal control. *P < 0.05, **P < 0.01, ***P < 0.001.

**Figure 3:**
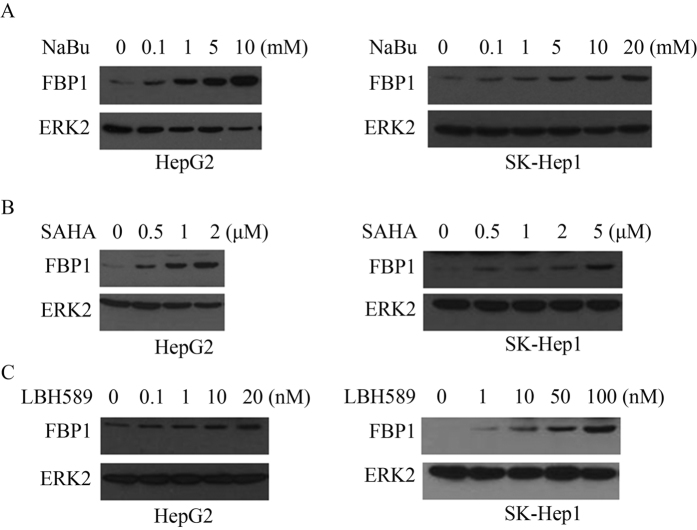
Effects of HDAC inhibitors on FBP1 protein expression in HCC cells. HepG2 and SK-Hep1 cells were treated with different dosage of HDAC inhibitors including NaBu (**A**), SAHA (**B**) and LBH589 (**C**) for 24 hours. Cells were harvested and FBP1 protein was analyzed by western blotting. ERK2 protein was used as a loading control. Cropped gels were displayed and full-length gels and blot were included in the Supplementary Information file.

Our data obtained from clinical specimens suggest that decreased expression of FBP1 favors HCC progression. We were very interested to determine whether we could restore FBP1 expression in HCC cells and whether restored expression of FBP1 could inhibit HCC cell growth. Since histone deacetylation generally correlates with gene repression, we sought to determine whether treatment of HCC cells with HDAC inhibitors could cause FBP1 derepression. Butyrate, a four-carbon short-chain fatty acid product of fiber fermentation within the colon, is a naturally occurring HDAC inhibitor^14^. We demonstrated that treatment of HCC cell lines HepG2 and SK-Hep1 with sodium butyrate (NaBu) induced expression of *FBP1* mRNA in a dose-dependent manner (Fig. 2A). To further validate this observation, we treated both HepG2 and SK-Hep1 cell lines with two other HDAC inhibitors suberoylanilide hydroxamic acid (SAHA or vorinostat) and LBH589 (or panobinostat). Similar to the effect of NaBu, SAHA and LBH589 treatment also induced expression of *FBP1* mRNA in both cell lines in a dose-dependent manner (Fig. 2B and C). Similar to the effect on mRNA, treatment of both cell lines with three HDAC inhibitors invariably increased expression of FBP1 proteins in a dose-dependent manner (Fig. 3). Thus, these data suggest that HDAC inhibitors can restore the expression of FBP1 mRNA and protein in human HCC cells.

### HDAC1 and HDAC2 work in concert to repress FBP1 expression in HCC cells

**Figure 4:**
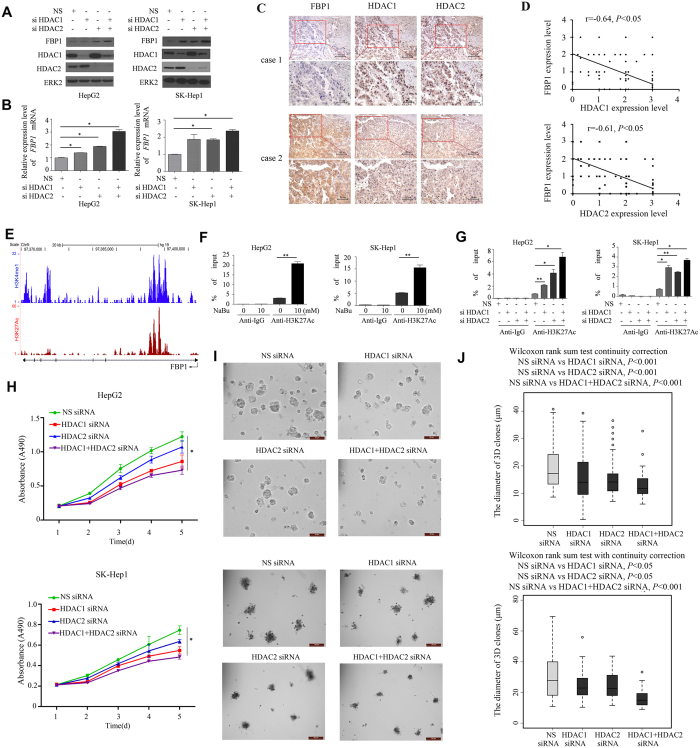
HDAC1 and HDAC2 inhibit FBP1 expression, decrease H3K27 acetylation in the *FBP1* enhancer, and promote HCC cell proliferation. (**A**) Western blot analysis of FBP1 expression in HepG2 and SK-Hep1 cells transfected with control or pool of HDAC1 and/orHDAC2-specific siRNAs. ERK2 protein was used as a loading control. (**B**) Real-time RT-PCR analysis of *FBP1* mRNA expression in cells transfected with control or pool of HDAC1 and/orHDAC2-specific siRNAs. *GAPDH* was used as an internal control. (**C**) IHC staining of FBP1, HDAC1 and HDAC2 proteins in a cohort of HCC patient samples. (**D**) Correlation analysis of the staining index (SI) for expression of FBP1 and HDAC1 or HDAC2 proteins in human HCC specimens (n = 90). Nonparameter Spearman correlation co-efficiency and the *P* values are also shown. (**E**) Screen shots from the UCSC genome browser displaying signal peak of H3K27Ac binding (ChIP-seq) in prostate cancer cells as reported previously^36^. The ChIP-seq signaling for the enhancer mark H3K4me1^17^ is also included. (**F**) Real-time PCR analysis of DNA immunoprecipitated by control IgG or H3K27Ac antibody from HepG2 and SK-Hep1 cells treated with the HDAC inhibitor NaBu. Cells harvested for ChIP assay at 24 hours after treatment. (**G**) Real-time PCR analysis of DNA immunoprecipitated by control IgG or H3K27Ac antibody from HepG2 and SK-Hep1 cells transfected with control or HDAC1- and/or HDAC2-specific siRNAs. Cells harvested for ChIP assay at 48 hours after transfection. (**H**) HepG2 and SK-Hep1 cells were transiently transfected with NS siRNA control or HDAC1 and/or HDAC2-specific siRNAs. After 24-hour transfection, cell growth was determined by measuring A490 absorbance at different time points. Data shown as means ± SD (n = 6 biological replicates). (**I**) The representative images of 3D cultures of HCC cells transfected with NS siRNA control, HDAC1 and/or HDAC2-specific siRNAs at day 5. Scale bar, 50 μm. (**J**) The diameter of 3D clones (n ≥ 50) at day 5 was measured by Leica software (LAS V4.2). Differences in the mean diameters of 3D clones between cells transfected with NS siRNA control or HDAC1 and/or HDAC2 siRNA were compared using Wilcoxon rank sum test with continuity correction (R software). *P < 0.05, **P < 0.01. Cropped gels were displayed and full-length gels and blot were included in the Supplementary Information file.

To delineate which HDAC family member(s) play an important role in repressing FBP1 expression in HCC cells, we focused our attention on HDAC1 and HDAC2 among the 18 different members of the family because they are often deregulated in human cancers. HepG2 and SK-Hep1 cells were transfected with non-specific (NS), pool of HDAC1-specific and/or HDAC2-specific siRNAs. We demonstrated that knockdown of HDAC1 or HDAC2 individually led to a small increase in FBP1 expression at both the protein and mRNA levels (Fig. 4A and B). Importantly, knockdown of HDAC1 and HDAC2 together resulted in a much greater elevation of FBP1 mRNA and protein in both cell lines (Fig. 4A and B). This result is consistent with the drug treatment data as described above. We conclude that HDAC1 and HDAC2 work together to repress FBP1 expression in HCC cells. However, our data cannot rule out the possibility that other HDAC family members may also contribute to the downregulation of FBP1 in HCC cells and therefore further investigation is warranted.

### FBP1 expression inversely correlates with HDAC1 and HDAC2 expression in HCC patient specimens

The finding that HDAC1 and HDAC2 repress FBP1 expression in cultured HCC cells prompted us to determine whether expression of HDAC1 and HDAC2 inversely correlates with FBP1 levels in clinical samples. We analyzed FBP1, HDAC1 and HDAC2 protein expression in the same patient samples using immunohistochemistry (IHC). We demonstrated that FBP1 was expressed in the cytoplasm and nucleus while HDAC1 and HDAC2 were primarily localized in the nucleus (Fig. 4C). Furthermore, correlation analysis indicated that FBP1 expression inversely correlated with HDAC1 and HDAC2 proteins in a cohort of 90 HCC patients (r = −0.64, *P* < 0.05; r = −0.61, *P* < 0.05) (Fig. 4D).

### HDAC1 and HDAC2 regulate H3K27Ac levels in a putative *FBP1* enhancer

As HDAC inhibitor treatment and knockdown of HDAC1 and HDAC2 invariably lead to FBP1 upregulation, next we were interested to decipher the molecular mechanism of HDAC-mediated repression of FBP1 in HCC cells. We hypothesized that HDAC proteins may repress FBP1 expression by regulating the histone acetylation levels in its enhancer/promoter. Histone H3 lysine 4 monomethylation (H3K4Me1) is a typical marker of enhancers^15^,^16^. We noticed that there is a putative H3K4me1-positive enhancer region in the first intron of the *FBP1* gene (Fig. 4E). Importantly, we found that at the same region there is a peak of histone H3 lysine-27 acetylation (H3K27Ac) (Fig. 4E), the level of which often correlates with transcriptional activity of the gene. We first examined whether treatment of the HDAC inhibitor NaBu affects H3K27Ac levels in this region. To this end, we performed chromatin immunoprecipitation (ChIP) assays using H3K27Ac-specific antibody. As demonstrated in Fig. 4F, the H3K27Ac level was readily detectable in the enhancer region of *FBP*1 gene in both HepG2 and SK-Hep1 cell lines. However, consistent with the finding that NaBu treatment markedly increased expression of *FBP1* mRNA (Fig. 2A), it also significantly increased H3K27Ac level in the *FBP1* enhancer (Fig. 4F). Similar to the finding that HDAC1 and HDAC2 knockdown induced FBP1 expression, depletion of these proteins alone or together also largely increased H3K27Ac level at this locus (Fig. 4G). This data indicate that the *FBP1* gene locus indeed harbors a regulatory region where H3K27Ac level can be modulated by HDAC proteins. Thus, HDAC inhibitor-induced upregulation of H3K27 acetylation in the enhancer of the *FBP1* gene correlates with increased expression of FBP1 induced by HDAC inhibitors.

Next, we examined the effect of HDAC1 and HDAC2 on proliferation of HCC cells. To this end, we knocked down HDAC1 or HDAC2 alone or both and performed both MTS and three dimension (3D) matrigel assays. We demonstrated that knockdown of HDAC1, HDAC2 or both significantly suppressed growth of both HepG2 and SK-Hep1 cell lines (Fig. 4H–J). These data indicate that inhibiting histone deacetylases suppresses HCC cell growth.

### Sodium butyrate decreases glucose reduction, lactate secretion and cell proliferation via modulating FBP1 expression

**Figure 5:**
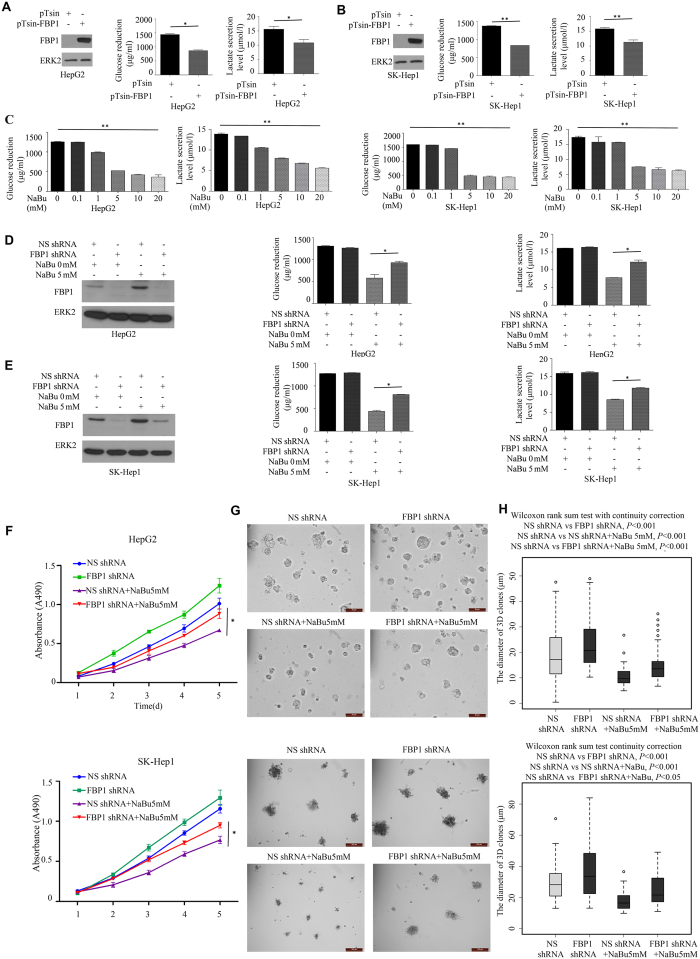
Restored expression of FBP1 decreases glucose reduction, lactate secretion and cell proliferation. (**A**,**B**) Glucose reduction (original level minus remaining amount) and lactate secretion were determined in the spent medium of HepG2 cells (**A**) and SK-Hep1 cells (**B**) infected with control lentiviral vector pTsin or pTsin-FBP1 and cultured for 48 hours. (**C**) Glucose reduction and lactate secretion were determined in the spent medium of HepG2 cells and SK-Hep1 cells treated with different concentrations of the HDAC inhibitor NaBu. (**D**,**E**) HepG2 (**D**) and SK-Hep1 (**E**) cells were infected with non-specific control or FBP1-specific shRNAs for 48 hours and then treated with NaBu at 0 or 5 mM for 24 hours and then expression of FBP1 protein and glucose reduction and lactate levels were determined. (**F**) HepG2 and SK-Hep1 cells infected with lentivirus for non-specific (NS) shRNA or FBP1-specific shRNA were treated with or without NaBu (5 mM). Cell growth was determined by measuring A490 absorbance at different time points. Data shown as means ± SD (n = 6 biological replicates). (**G**) The representative images of 3D clones of HepG2 and SK-Hep1cells infected with lentivirus for NS shRNA control or FBP1-specific shRNA and treated with or without NaBu (5 mM) at day 5. Scale bar, 50 μm. (**H**) Differences in the mean diameters of 3D clones (n ≥ 50) at day 5 was compared between cells infected with NS shRNA control or FBP1-specific shRNA and treated with or without NaBu (5 mM). Differences were compared using Wilcoxon rank sum test with continuity correction (R software). *P < 0.05, **P < 0.01. Cropped gels were displayed and full-length gels and blot were included in the Supplementary Information file.

Since FBP1 is the rate-limiting enzyme in regulating glucose homeostasis^17^, we sought to determine whether restored expression of FBP1 decreases glucose reduction and lactate secretion in HCC cells. We first ectopically expressed FBP1 in HepG2 and SK-Hep1 cells. We demonstrated that forced expression of FBP1 significantly diminished glucose reduction and lactate secretion in both cell lines (Fig. 5A and B).

We next explored if HDAC inhibitor affects glucose metabolism and lactate secretion in HCC cells. HepG2 and SK-Hep1 cells were treated with different doses of NaBu, and glucose reduction and lactate secretion were determined. We demonstrated that NaBu treatment mitigated glucose reduction and lactate secretion in a dose-dependent fashion in both cell lines (Fig. 5C). To determine the role of FBP1 in the regulation of glucose reduction and lactate secretion by NaBu, we depleted FBP1 expression in these cells by infecting cells with non-specific (NS) or FBP1-specific shRNA prior to NaBu treatment. To our surprise, the effect of FBP1 knockdown alone on glucose reduction and lactate secretion was negligible in both cell lines (Fig. 5D and E). A plausible explanation is that the basal levels of FBP1 are relatively low in these cell lines and their role in leveraging glucose level and lactate secretion can be limited. As expected, NaBu treatment increased FBP1 expression, which is consistent with decreased glucose reduction and lactate secretion in these cells (Fig. 5D and E). Importantly, NaBu-induced decrease in glucose reduction and lactate secretion were largely diminished by FBP1 knockdown (Fig. 5D and E). It worth noting that although FBP1 protein was almost completely depleted (at least the case in HepG2 cells), FBP1 depletion failed to completely block the effect of NaBu on glucose reduction and lactate secretion, suggesting that NaBu can also regulate glucose level and lactate secretion through FBP1-independent mechanisms. Nevertheless, we demonstrate that HDAC inhibition can suppress glucose reduction and lactate secretion and such effect is mediated, at least in part, through HDAC inhibitor-induced upregulation of FBP1 in HCC cells.

We also examined the role of FBP1 in the regulation of HCC cell growth by the HDAC inhibitor NaBu using both MTS and matrigel assays. We depleted FBP1 expression in HepG2 and SK-Hep1 cells by infecting cells with non-specific (NS) or FBP1-specific shRNA prior to NaBu treatment. Knockdown of FBP1 increased proliferation of both HepG2 and SK-Hep1 cells (Fig. 5F–H). Moreover, NaBu treatment decreased proliferation in both cell lines, but this effect was partially reversed by FBP1 knockdown (Fig. 5F–H). Together, our data indicate that HDAC inhibition can suppress HCC cell proliferation, at least in part, through HDAC inhibitor-induced upregulation of FBP1.

### Restored expression of FBP1 inhibits HCC cell growth *in vitro* and in mice

**Figure 6:**
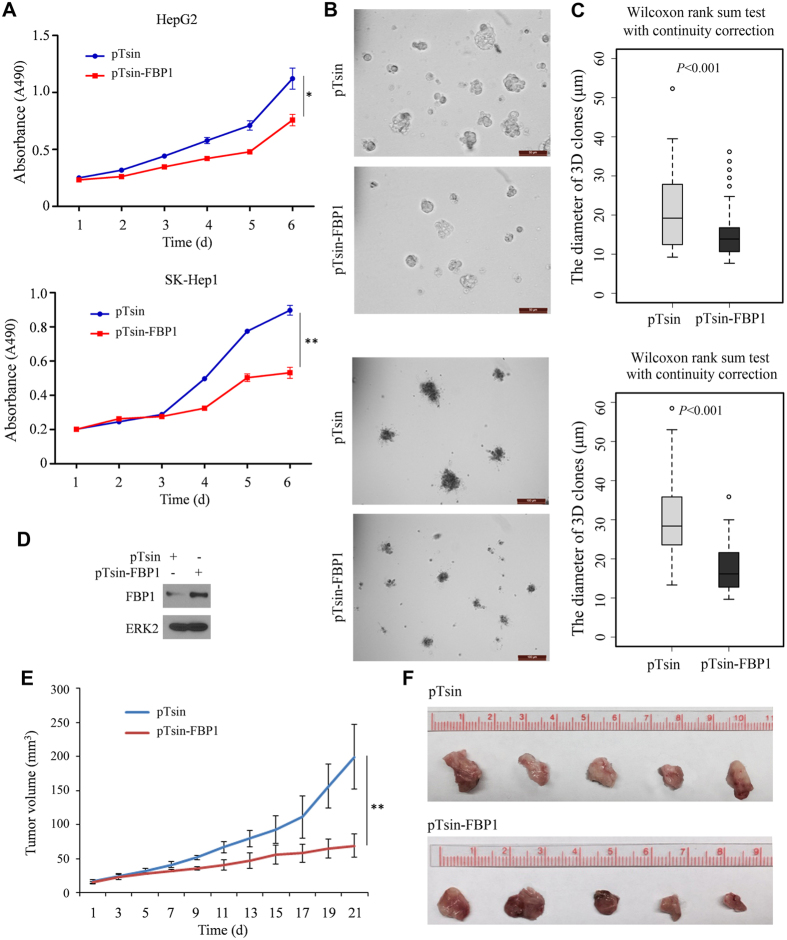
Restored expression of FBP1 inhibits HCC cell growth. (**A**) Growth of HepG2 and SK-Hep1 cells infected with lentivirus for control vector pTsin or pTsin-FBP1 were measured at different time points using MTS assay. (**B**) The representative images of 3D clones of HepG2 and SK-Hep1 cells infected with lentivirus for control vector pTsin control or pTsin-FBP1 for 5 days. Scale bar, 50 μm. (**C**) Differences in the mean diameters of 3D clones (n ≥ 50) at day 5 were compared between cells infected with lentivirus as indicated. *P* value was calculated using Wilcoxon rank sum test with continuity correction (R software). (**D**–**F**) SK-Hep1 cells infected with control lentiviral vector pTsin and pTsin-FBP1 were injected subcutaneously into NSG mice (n = 5/group). Expression of FBP1 was measured by western blotting and ERK2 was used as a loading control (**D**). Tumor volume were measured at different time points (**E**) and the tumors at day 21 were photographed (**F**). *P < 0.05, **P < 0.01. Cropped gels were displayed and full-length gels and blot were included in the Supplementary Information file.

Given that cancer cells rely heavily on aerobic glycolysis (the Warburg effect) for cell growth, we sought to determine whether restored expression of FBP1 inhibits HCC cell growth. We transduced control or FBP1 lentiviral expression vector into HepG2 and SK-Hep1 cells. We demonstrated that restored expression of FBP1 substantially inhibited growth of these cell lines in culture (Fig. 6A). Similar results were obtained in 3D matrigel assays in both cell lines (Fig. 6B and C). Next, we assessed the effect of the restored expression of FBP1 on tumor growth in mice. We inoculated control or FBP1-infected SK-Hep1 cells in NOD-SCID IL-2-receptor gamma null (NSG) mice. We found that increased expression of FBP1 significantly inhibited tumor growth *in vivo* (Fig. 6D–F). Therefore, our data demonstrate that restored expression of FBP1 inhibits HCC cell growth both *in vitro* and *in vivo*.

## Discussion

FBP1 is the rate-limiting enzyme of gluconeogenesis. FBP1 and phosphofructokinase maintain the equilibrium between fructose-6-phosphate and fructose-1,6-diphosphate. Loss of FBP1 has been reported in several cancers^5^,^6^,^7^,^8^. Its expression in tumors is significantly lower than that in non-tumor tissues, as confirmed in gastric cancer cell lines and others^6^,^7^,^18^,^19^. Restoration of its expression in cancer cells can suppress glycolysis and inhibit tumor cell proliferation. The methylation of FBP1 promoter has been observed to be associated with gastric TNM stage and patients’ survival, and it is an independent prognostic factor for gastric cancer^6^. Also, loss of FBP1 expression in renal clear cell carcinoma associates with patient prognosis^8^. It seems to inhibit the Warburg effect of cancer by inhibiting glycolysis in kidney tubular epithelial cells. In breast cancer cells, its loss promotes glycolysis, resulting in increased glucose uptake, increased PKM2 tetramer formation, and enhanced hypoxia-dependent ATP production^5^. FBP1 has also been observed to reduce oxygen consumption and ROS production through inhibition of mitochondrial complex I activity. The metabolic changes due to FBP1 loss could increase tumorigenicity of breast cancer cells^5^. Liver is an important organ for gluconeogenesis. Our data and those from TCGA show that FBP1 is highly expressed in tumor-adjacent benign tissues. It has been reported previously that FBP1 expression is downregulated in HCC patient samples and cell lines^20^,^21^. In our study, we first examined FBP1 expression level in 90 HCC and adjacent benign tissues. Consistent with the finding of previous studies^20^,^21^, we demonstrated that FBP1 protein level in HCC tissues was significantly lower than that in adjacent benign tissues in both the cohort of HCC patients and in the TCGA dataset. Most importantly, we demonstrate that low expression of FBP1 associated with the tumor size and the TNM stage of HCC. Furthermore, the average survival time and the cumulative survival time of the FBP1-high-expression group were significantly higher than that of the FBP1-low-expression group. These findings are highly significant since they suggest that FBP1 not only plays an essential role in facilitating HCC tumor progression, but also can be a therapeutic target of HCC.

The role of epigenetic gene regulation in the development of cancer has been well recognized. Histone modification is an important form of epigenetic regulation. HDACs are a group of enzymes that are critical in regulation of chromatin structure and gene expression. HDAC family members have been observed to be overexpressed in gastric cancer, lung cancer and other tumors^22^,^23^,^24^,^25^. Currently, the regulatory mechanism of FBP1 transcription and translation in HCC remains poorly understood. ChIP-seq profiles in the public datasets indicate that there is a putative H3K27Ac site in the *FBP1* enhancer, implying a role of H3K27Ac regulators (e.g. HDACs and histone acetyltransferase (HATs)) in modulation of FBP1 expression in HCC cells.

There are four kinds of HDAC inhibitors approved by the Food and Drug Administration (FDA) of USA for cancer therapy in clinic^26^. Sodium butyrate (NaBu) is one of the first discovered HDAC inhibitor. It promotes the expression of *p21*^*CIP1*^ gene by increasing the relevant chromatin histone acetylation level so that the cell cycle is arrested at the G1 stage, thereby inhibiting proliferation of cancer cells. Animal experiments have further confirmed that NaBu can inhibit the tumor development in nude mice^27^. SAHA, another HDAC inhibitor, induces apoptosis of CTCL cells by accumulating histone acetylation, increasing expression of *p21*^*CIP1*^ and *bax*, decreasing of Stat6 and phospho-Stat6 proteins, activating caspase-3 and cleaving PARP^28^. It is the first HDAC inhibitor approved by FDA and used for clinical treatment of cutaneous T cell lymphoma^28^. LBH589 is a hydroxamic acid that inhibits cell proliferation and induces cell apoptosis in lung and gastric cancer^29^,^30^. It inhibits the invasion and migration of breast cancer cells by inducing the expression of E-cadherin without affecting the estrogen pathway^31^. In addition, there are several other kinds of HDAC inhibitors, but we focused on these three HDAC inhibitors. The protein and mRNA levels of FBP1 were significantly increased after cells were treated with these inhibitors. We further tested if this mechanism was involved in histone acetylation. The results of ChIP analysis showed that H3K27 acetylation levels in the *FBP1* enhancer increased after treatment with HDAC inhibitors. Our experiments confirmed that HDAC inhibitors upregulated the FBP1 protein and mRNA expression, which correlates with increased H3K27Ac level at the *FBP1* enhancer. In addition, there are many subtypes of HDACs such as class I HDAC, which is primarily related to the proliferation of tumor cells and class II HDAC that predominantly is tissue-specific. HDAC1 and HDAC2 are known as the classic I histone deacetylases. They play an important role in maintaining the acetylation status of nucleosome. Our data showed that knocking down HDAC1 and HDAC2 individually led to mild elevation of FBP1 protein and mRNA expression levels, but simultaneous knockdown of them significantly increased FBP1 expression in HCC cells. The ChIP-qPCR results further confirmed the involvement of HDAC1 and HDAC2 in modulation of H3K27 acetylation at the *FBP1* enhancer and regulation of FBP1 expression. Importantly, this was further validated by IHC results that FBP1expression inversely correlates with HDAC1and HDAC2 protein level in human HCC tissues.

Based on the Warburg theory, tumor cells produce energy through glycolysis despite having sufficient oxygen supply. This change in energy metabolism is believed to associate with tumor development. Therefore, it has been proposed that interference in the glycolytic pathway of tumor cells may provide a way to treat cancer^32^. FBP1 is the rate-limiting enzyme that can block the glycolysis, by converting 1,6-fructose diphosphate to fructose-6-phosphate. In this study we also analyzed the glucose level and lactate secretion in the presence or absence of FBP1. Our data showed that restoration of FBP1 decreased glucose reduction and lactate secretion and inhibited tumor cell growth. Our results are consistent with the findings in the two most recent reports showing that FBP1 downregulation in HCC contributes to tumor progression and poor prognosis by altering glucose metabolism^20^,^21^. We further showed that HDAC inhibitors can also inhibit the ability of cancer cells to consume glucose and lactate secretion by upregulating FBP1 expression. Besides glucose metabolism, we also demonstrated that FBP1 could inhibit HCC cell proliferation and tumor growth in mice. Dexamethasone is an active form of synthesized glucocorticoids. It can restore gluconeogenesis in malignant cells, thereby leading to therapeutic efficacy in the treatment of hepatocarinoma^33^. In the present study we demonstrated that restoring the expression of FBP1 in hepatocellular carcinoma cells can also switch glycolysis to gluconeogenesis, alter energy metabolism in HCC cells and inhibit tumor growth. Thus, similar to the strategy of utilization of glucocorticoids, restoration of FBP1 expression by HDAC inhibitors might be harnessed for HCC therapy.

In conclusion, our study demonstrates that FBP1 expression is downregulated in a cohort of HCC tissues and this result is further validated by meta-analysis of the TCGA dataset. The association of low FBP1 expression with several clinicophathological parameters suggests that loss or decreased expression of FBP1 can be an independent prognostic factor for HCC patients. Mechanistically, we demonstrate that FBP1 expression is regulated by H3K27 acetylation through a region in its enhancer and that pharmacological inhibition of HDACs and genetic depletion of HDAC1 and HDAC2 increases H3K27Ac level and induce FBP1 derepression. This observation is further validated by our finding in human HCC tissues that low expression of FBP1 expression correlates with high levels of HDAC1 and HDAC2. Additionally, we demonstrate that restoration of FBP1 expression in HCC cells blocks glycolysis and inhibits tumor growth. Similarly, HDAC inhibitors can also achieve a similar effect by up-regulating FBP1 expression in HCC cells. Therefore, decreased expression of FBP1 can be targeted by HDAC inhibitors for potential treatment of HCC.

## Materials and Methods

### Tissue microarray and immunohistochemistry

The tissue microarray (TMA) slides were purchased from Shanghai Outdo Biotech Co. Ltd (Lot No. HLiv-HCC180Sur-02). The TMA slides contained ninety HCC and adjacent tissues collected from patients who underwent surgical resection between August 2006 and November 2009. All these specimens were diagnosed to be HCC and at tumor-node-metastasis stage by two pathologists independently. All these cases were followed up for 4–6 years. FBP1 mRNA expression data and the related clinical information were downloaded from hepatocellular carcinoma provisional dataset in The Cancer Genome Atlas (TCGA) (http://tcga-data.nci.nih.gov/). The study protocol was performed in accordance with the guidelines outlined in the Declaration of Helsinki and was approved by the Ethics Committee of Third Affiliated Hospital of Soochow University. Written informed consent was obtained from all participants.

The TMA slides were dewaxed in xylene, and rehydrated in graded ethanol solution. Next, the tissues were incubated in 0.3% H_2_O_2_ for 20 min to block endogenous peroxidase activity. Antigen retrieval was accomplished by boiling the tissues at 100 °C for 20 min in antigen retrieval solution. After blocking for 1 hour, tissues were incubated with primary antibodies at 4 °C overnight (FBP1 antibody: Sigma-Aldrich, St, Louis, MO, 1:4,000; HDAC1: Santa Cruz Biotechnology, CA, 1:500; HDAC2: Santa Cruz Biotechnology, CA, 1:500). The sections were incubated with anti-mouse or -rabbit secondary antibody for 1 hour at room temperature, followed by further incubation with ABC solution for 30 min and DAB solution for chromogen detection and haematoxylin solution for nuclear stain. Xylene and graded ethanol solution were used for dehydration.

The IHC staining was scored by two independent pathologists. Based on the percentage of positive cells, the staining was scored as follows: score 0, no positive cells; score 1, 1~25 percent of positive cells; score 2, 26~50 percent of positive cells; score 3, >50 percent of positive cells. The criteria for the staining intensity were as follows: 0 for negative; 1 for low staining; 2 for media staining; 3 for strong staining. The final staining index (SI) for each staining was obtained by multiplying values obtained from staining percentage and intensity.

### Cell culture and treatment

HCC cell lines HepG2 and SK-Hep1 were purchased from the American Type Culture Collection (ATCC, Manassas, VA, USA) and were cultured at 5% CO_2_, 37 °C and 95% humidity. HepG2 cells were cultured in DMEM medium containing with 10% fetal bovine serum (Thermo Fisher Scientific) and 100 units/ml penicillin and 100 μg/ml streptomycin (Thermo Fisher Scientific). SK-Hep1 cells were cultured in EMEM medium supplemented with 10% fetal bovine serum and 100 units/ml penicillin and 100 μg/ml streptomycin. In glucose level measurement experiments, both cell types were grown in RPMI 1640 medium without phenol red and containing 10% fetal bovine serum and 100 units/ml penicillin and 100 μg/ml streptomycin. For chemical treatment experiments, cells were treated with various concentrations of sodium butyrate (NaBu), SAHA (Sigma-Aldrich, St. Louis, MO) or LBH589 (Selleckchem, Houston, TX).

### Western blot analysis

Cells were lysed by incubation in RIPA lysis buffer [1xPBS, 1% Nonidet P-40, 0.1% sodium dodecyl sulfate and protease inhibitor cocktail (Sigma-Aldrich, St. Louis, MO)]. The protein concentrations were measured by BCA method, and then samples were diluted in loading buffer containing DTT and boiled for 5 min. Equal amount of protein for each sample was separated by 10% SDS-polyacrylamide gels and transferred onto nitrocellulose membranes. Subsequently, membranes were incubated with primary antibodies against FBP1 (1:500), HDAC1 (1:1,000), HDAC2 (1:1,000) and ERK2 (1:8,000) overnight at 4 °C. Next day, the membranes were washed with 1xTBST and incubated at room temperature with HRP-conjugated secondary antibodies for 1 hour. Finally, proteins were detected by chemiluminescence.

### RNA extraction and real-time RT-PCR

Total RNA was extracted from cells by using Trizol reagent (Thermo Fisher Scientific). cDNA was synthesized by using Superscript II reverse transcriptase (Thermo Fisher Scientific). Real-time PCR was performed by using IQ SYRB Green Supermix and an iCycleriQTX detection system (Bio-Rad). All the signals were normalized against *GAPDH* and the 2-△Ct method was used to quantify the fold change. The following primers were used: *FBP1*, forward 5′-ACATCGATTGCCTTGTGTCC-3′ and reverse 5′-CCACCAAAATGAACTCCCCG-3′; *GAPDH*, forward 5′-ACCCACTCCTCCACCTTTGAC-3′ and reverse 5′-TGTTGCTGTAGCCAAATTCGTT-3′.

### Cells transfection

Transfections were performed using either electroporation method with an Electro Square Porator ECM 830 (BTX)^34^ or Lipofectamine 2000 (Thermo Fisher Scientific). Small interfering RNA (siRNA) specific for HDAC1, HDAC2 and nonspecific (NS) control were purchased from GE Health Care-Dharmacon (Lafayette, CO). Cells were collected 48 hours after transfection and approximately 75–90% transfection efficiencies were routinely achieved. FBP1 overexpression plasmid was transfected by using Lipofectamine 2000 and empty plasmid was used as control.

### Chromatin immunoprecipitation (ChIP) assay

HepG2 and SK-Hep1 cells were treated with sodium butyrate (NaBu) 10 mM for 24 hours or transfected with HDAC1 siRNA, HDAC2 siRNA or both. ChIP assay was performed as described earlier^35^. The chromatin samples were incubated with 5 μg antibodies against IgG (Santa Cruz Biotechnology, CA) or H3K27 acetylation (Abcam, Cambridge, UK). Primers specific for H3K27 acetylation peak region in the *FBP1* enhancer were: forward 5′-TCTCCTCCCGAAGTCACTGT-3′ and reverse 5′-ATCTCCCTCCCTTTTGGTTG-3′. Real-time PCR was performed by using IQ SYRB Green Supermix and an iCycleriQTX detection system (Bio-Rad).

### Glucose and lactate measurement

Cells were cultured in 6-well plates for 24 hours (4.0 × 10^5^/well), and cultured with RPMI 1640 medium without phenol red and treated with different concentration of sodium butyrate (NaBu) or transfected with FBP1 shRNA or pTsin-FBP1. Culture supernatants were collected after 48 hours. Glucose concentration was measured according to the instructions of the test kit (Sigma-Aldrich, St. Louis, MO). Lactate concentration was measured according to the instructions of the test kit (Eton Bioscience). The optical densities were measured at 570 nm wave length.

### Three-dimension (3D) matrigel culture

One hundred twenty micro-liter of matrigel was applied onto the bottom of 24-well plate and incubated in an incubator at 37 °C to generate the first layer. Following 30-min incubation, approximately 25,000 HepG2 or SK-Hep1 cells suspended in 250 μl of DMEM: F12 medium without serum and seeded on the top of the first layer, and then 250 μl of 10% Matrigel diluted with DMEM: F12 medium was applied on the top of cells. 500 μl of DMEM: F12 + 5% FBS + P/S medium was added and changed every two to three days. The pictures were taken with Leica microscope at day 5, and the diameter of 3D clones (n > 50) was measured with Leica software (LAS V4.2).

### MTS assay

HepG2 and SK-Hep1 cells (2 × 10^3^/well), stably transfected with lentivirus for empty vector (pTsin), pTsin-FBP1 or FBP1 shRNA, were cultured in 96-well plates and treated with or without NaBu. At indicated time points, the 20 μl of MTS solution (Promega) was added into each well, and the plate was incubated at 37 °C for 2 hours. The absorbance was measured at wave length of 490 nm.

### Xenograft studies

6-week-old NOD-SCID IL-2-receptor gamma null (NSG) mice were generated in house and used for animal experiments. The animal study and experiment protocols were approved by the IACUC at Mayo Clinic. The animals were maintained and handled in accordance with the Guidelines for Accommodation and Care of Animals. Jing Yang and Jingting Jiang have the license for animal experiments. All mice were housed in standard conditions of 12-hour light/dark cycle and access to food and water as libitum. SK-Hep1 cells (6 × 10^6^), infected with control (pTsin) or FBP1 expressing lentiviral vector (pTsin-FBP1), were injected subcutaneously into mice. Tumor size was measured and recorded every other day for 21 days and tumor volumes were calculated using the formula L × W^2^ × 0.5.

### Statistical analysis

All the data were analyzed by SPSS 13.0 software unless otherwise specified. Wilcoxon Signed Ranks Test was used to compare the expression level of FBP1 in tumor and adjacent tissues. The association of clinical parameters with the FBP1 expression level was evaluated by using chi-square test. Kaplan-Meier test and Log-rank test were used to determine the association of FBP1 expression with the overall patient survival. Differences between groups of three-dimension matrigel assays were determined using Wilcoxon rank sum test with continuity correction (R software). T-test was used to compare the mean values in most cell culture studies. *P* < 0.05 was considered statistically significant.

## Additional Information

**How to cite this article**: Yang, J. *et al*. Inhibiting histone deacetylases suppresses glucose metabolism and hepatocellular carcinoma growth by restoring FBP1 expression. *Sci. Rep.* **7**, 43864; doi: 10.1038/srep43864 (2017).

**Publisher's note:** Springer Nature remains neutral with regard to jurisdictional claims in published maps and institutional affiliations.

## Supplementary Material

Supplementary Information

## Figures and Tables

**Table 1:** Analysis of the correlation between FBP1 expression and clinicopathological data.

Clinical Characteristics	Case	FBP1 Expression		χ^2^	*P* value
		High (%)	Low (%)		
Sex					
men	78	27 (34.6)	51 (65.4)	0.008	0.931
women	12	4 (33.3)	8 (66.7)		
Age					
≤50	30	8 (26.7)	23 (73.3)	1.206	0.272
>50	60	23 (38.3)	36 (31.7)		
Tumor size					
≤3 cm	20	12 (60.0)	8 (40.0)	7.980	0.005
>3 cm	69	18 (26.1)	51 (73.9)		
Pathology stage					
I-II	56	23 (41.1)	33 (58.9)	2.883	0.090
III-IV	34	8 (23.5)	26 (76.5)		
AJCC stage					
I-II	43	22 (51.2)	19 (48.8)	10.471	0.001
III-IV	44	10 (18.2)	39 (81.8)		
Cirrhosis					
yes	33	13 (39.4)	20 (60.6)	0.565	0.452
no	57	18 (31.6)	39 (68.4)		

Some of the information is missing, including one case for tumor size and three cases for AJCC classification.

**Table 2:** Analysis of the relationship between FBP1 expression and patients’ cumulative survival rate.

Time (months)	Cumulative survival rate (%)	
	FBP1 low expression	FBP1 high expression
12	50.8	74.2
24	40.7	64.5
36	32.2	58.1
48	30.5	50.9
60	30.5	45.8
72	30.5	45.8
