# Supplementary material for: Inhibiting histone deacetylases suppresses glucose metabolism and hepatocellular carcinoma growth by restoring FBP1 expression
Source: Sci Rep. 2017 Mar 6;7:43864. doi: 10.1038/srep43864 (PMC5338333; doi:10.1038/srep43864)
Supplement: Supplementary Information [file srep43864-s1.pdf]

Inhibiting histone deacetylases suppresses glucose metabolism and hepatocellular carcinoma growth by restoring FBP1 expression

Jing Yang[1,2,4], Xin Jin[2], Yuqian Yan[2], Yingjie Shao[1,4], Yunqian Pan[2], Lewis Roberts[5], Jun Zhang[6], Haojie Huang[2,3*] and Jingting Jiang[1*]

[1]Department of Tumor Biological Treatment, The Third Affiliated Hospital of Soochow University, 185 Juqian Street, Changzhou 213003, China;
[2]Department of Biochemistry and Molecular Biology, Mayo Clinic College of Medicine, Rochester, MN 55905, USA;
[3]Mayo Clinic Cancer Center, Mayo Clinic College of Medicine, Rochester, MN 55905, USA;
[4]Jiangsu Engineering Research Center for Tumor Immunotherapy, Changzhou 213003, Jiangsu, China;
[5]Division of Gastroenterology and Hepatology, Mayo Clinic College of Medicine, Rochester, MN, USA;
[6]Department of Laboratory Medicine and Pathology, Mayo Clinic College of Medicine, Rochester, MN 55905, USA.

*__Corresponding authors__:
Haojie Huang, Ph.D. (huang.haojie@mayo.edu) and Jingting Jiang, M.D., Ph.D. (jiangjingting@suda.edu.cn).

Running title: Derepression of FBP1 by HDAC inhibitors

Full-length gels and blots in the figures were included in this supplementary information file. Aim gels were marked with the red frame.

Figure 3

HepG2

NaBu 0 0.1 1 5 10 (mM)

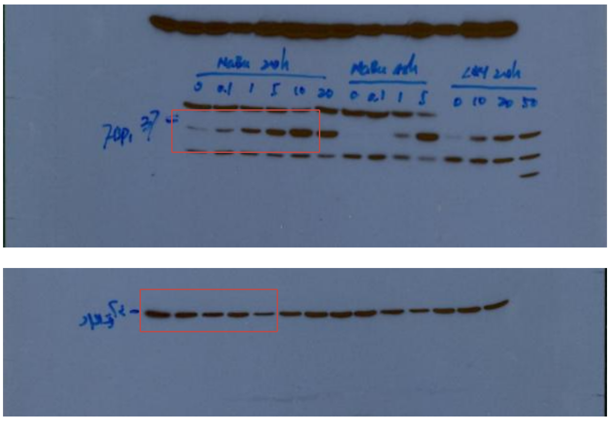

FBP1 -37

ERK2 -37

SAHA 0 0.5 1 2 (μM)

HepG2

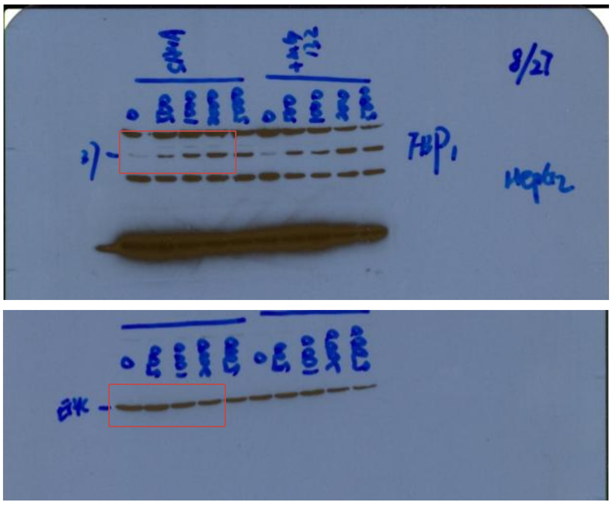

FBP1 -37

ERK2 -37

HepG2 LBH589 0 0.1 1 10 20(nM)

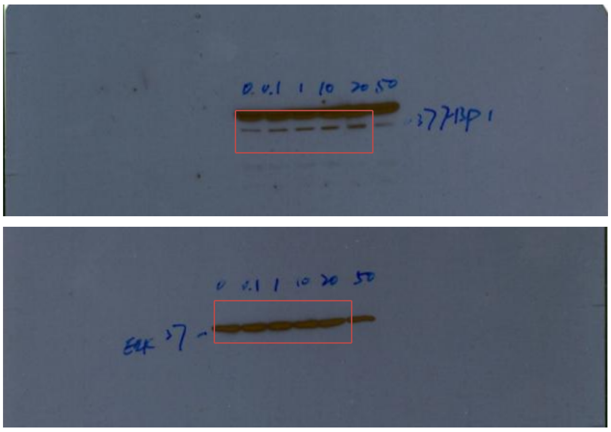

FBP1 -37

ERK2 -37

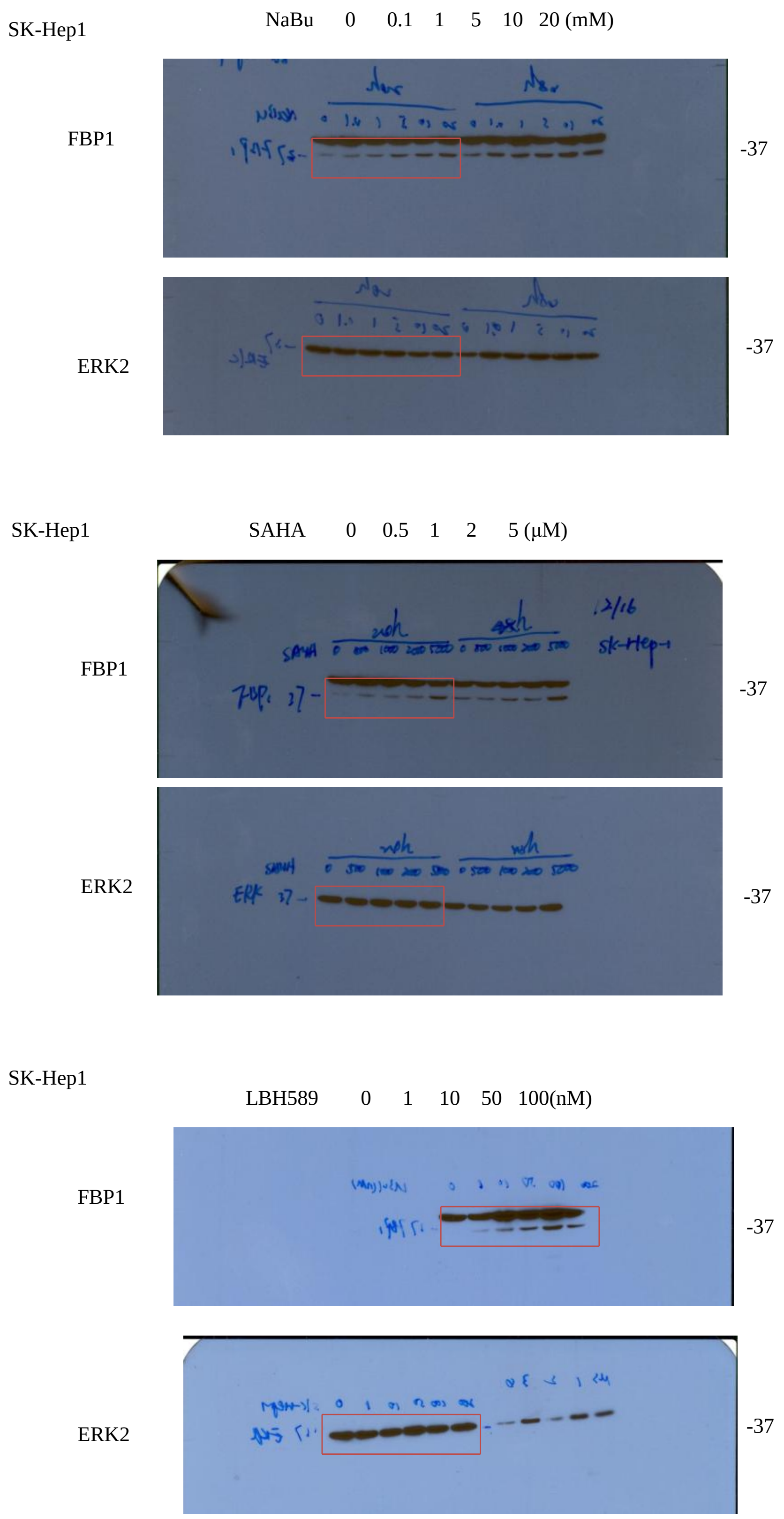
SK-Hep1
NaBu 0 0.1 1 5 10 20 (mM)
FBP1
-37
ERK2
-37
SK-Hep1
SAHA 0 0.5 1 2 5 (μM)
FBP1
-37
ERK2
-37
SK-Hep1
LBH589 0 1 10 50 100(nM)
FBP1
-37
ERK2
-37

Figure 4

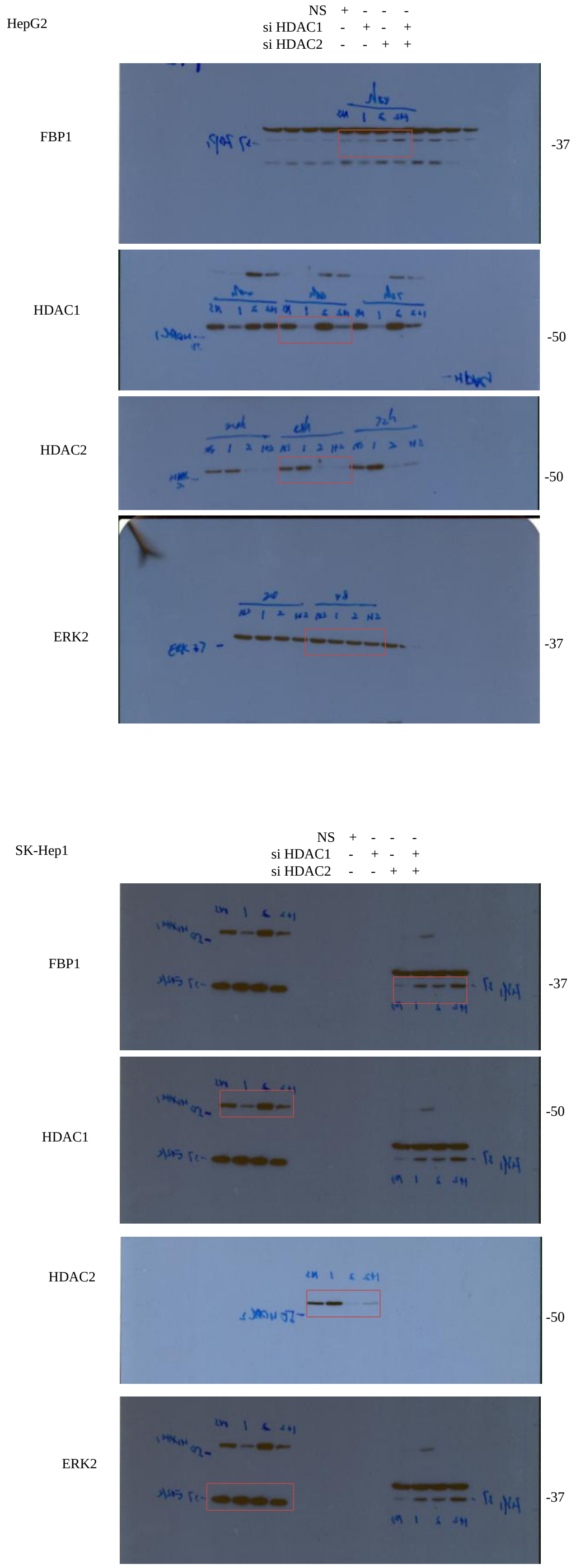
HepG2
NS + - - -
si HDAC1 - + - +
si HDAC2 - - + +
FBP1
-37
HDAC1
-50
HDAC2
-50
ERK2
-37
SK-Hep1
NS + - - -
si HDAC1 - + - +
si HDAC2 - - + +
FBP1
-37
HDAC1
-50
HDAC2
-50
ERK2
-37

Figure 5

HepG2

pTsin	+	-
pTsin-FBP1	-	+

FBP1 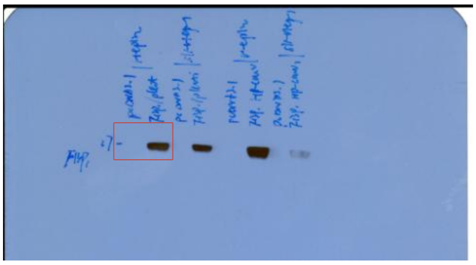 -37

ERK2 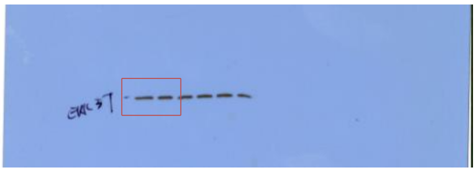 -37

SK-Hep1

pTsin	+	-
pTsin-FBP1	-	+

FBP1 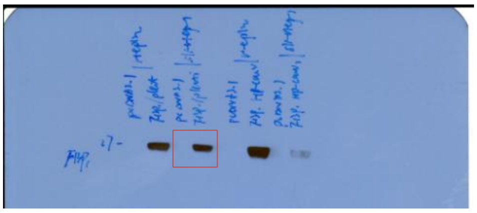 -37

ERK2 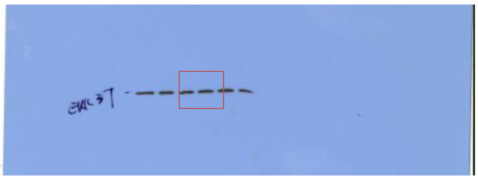 -37

HepG2

NS shRNA	+	-	+	-
FBP1 shRNA	-	+	-	+
NaBu 0mM	+	+	-	-
NaBu 5mM	-	-	+	+

FBP1 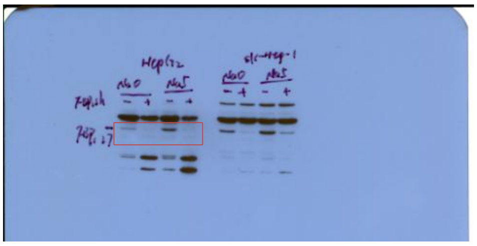 -37

ERK2 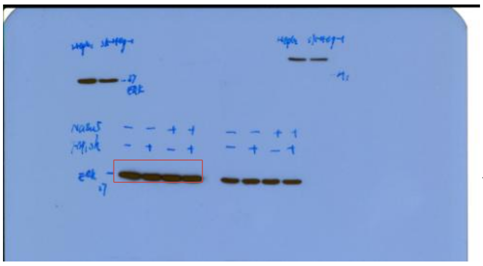 -37

SK-Hep1

NS shRNA	+	-	+	-
FBP1 shRNA	-	+	-	+
NaBu 0mM	+	+	-	-
NaBu 5mM	-	-	+	+

FBP1 -37

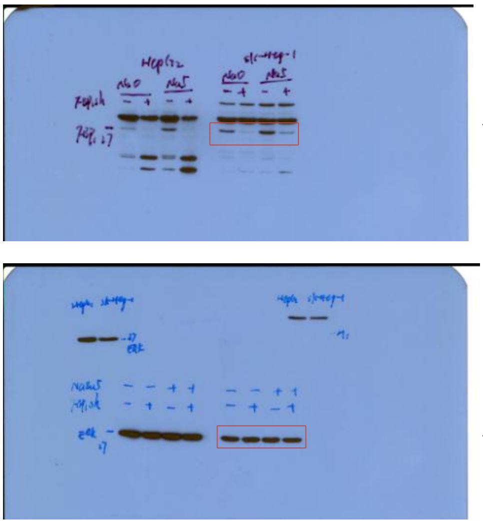

ERK2 -37

Figure 6

pTsin	+	-
pTsin-FBP1	-	+

FBP1 -37

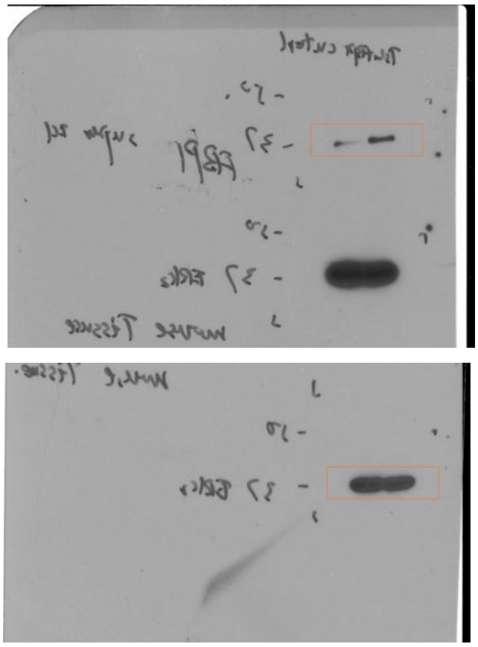

ERK2 -37

IHC data of Figure 4

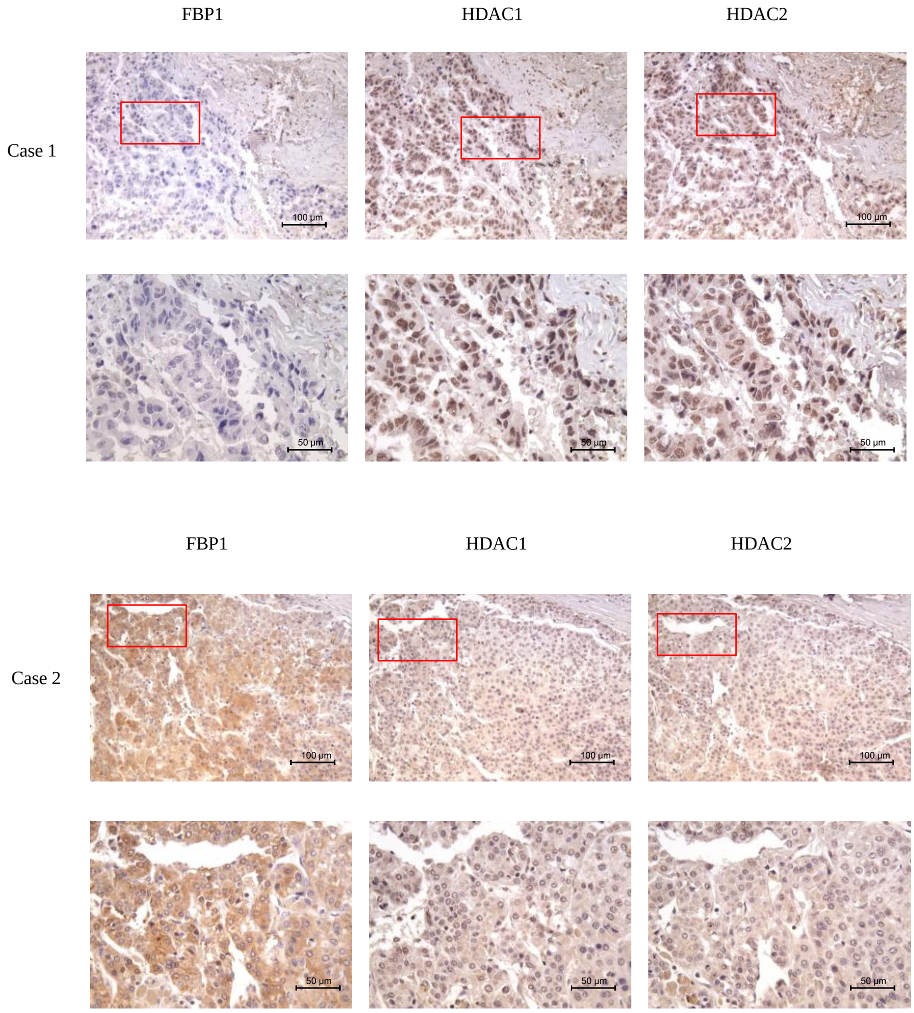
FBP1
HDAC1
HDAC2
Case 1
100 μm
50 μm
FBP1
HDAC1
HDAC2
Case 2
100 μm
50 μm
